# Diagnostic Tests for Stage B Heart Failure

**DOI:** 10.1007/s11886-026-02389-x

**Published:** 2026-07-04

**Authors:** Hai Nguyen Ngoc Dang, Thang Viet Luong, Quan Huynh, Thomas H. Marwick

**Affiliations:** 1 Menzies Institute for Medical Research, University of Tasmania, Hobart, Australia; 2 School of Medicine and Pharmacy, Duy Tan University, Da Nang, Vietnam; 3 Baker Heart and Diabetes Institute, Melbourne, Australia

**Keywords:** Stage B heart failure, Subclinical heart failure, Echocardiography, Cardiac biomarkers, Artificial intelligence

## Abstract

**Purpose of the Review:**

To provide an overview of diagnostic tests for Stage B heart failure (SBHF), synthesizing evidence from guidelines and clinical studies.

**Recent Findings:**

Advances in diagnostic technologies have expanded the ability to identify subclinical myocardial remodelling and early myocardial injury before symptom onset. We highlight the central role of transthoracic echocardiography as the cornerstone diagnostic modality for detecting subclinical myocardial remodelling and dysfunction, including the use of speckle tracking echocardiography. In parallel, circulating biomarkers, especially natriuretic peptides and high-sensitivity cardiac troponins, can play important roles in the detection and risk stratification of SBHF. Additional diagnostic approaches, including electrocardiography, chest X-ray, cardiac magnetic resonance imaging, cardiac computed tomography, nuclear imaging, and exercise stress testing, are reviewed for their adjunctive roles in selected clinical contexts. Emerging applications of artificial intelligence are explored as promising strategies to increase the diagnostic precision, scalability, and early detection of SBHF in clinical practice.

**Summary:**

SBHF – representing a subclinical phase of HF characterized by structural cardiac abnormalities, functional impairment, or persistently abnormal cardiac biomarkers in individuals – has historically been difficult to recognize in the community. Advances in imaging, biomarkers, and AI may improve the feasibility of detecting this entity, creating a crucial window for intervention, because timely risk stratification and preventive strategies during SBHF may attenuate progression to symptomatic HF and reduce its long-term clinical and economic burden.

**Supplementary Information:**

The online version contains supplementary material available at 10.1007/s11886-026-02389-x.

## Introduction

Heart failure (HF) remains a major global public health challenge, with a continuously increasing prevalence worldwide. This growing burden is largely driven by population aging and improved survival following acute cardiovascular events [1]. While it is historically a disease of elderly individuals, there is increasing concern about HF in younger people [2]. In response to this burden, major societies have issued regularly updated HF guidelines to optimize guideline-directed medical therapy, improve clinical outcomes, and reduce HF-related morbidity and mortality [3, 4]. However, despite broader implementation of evidence-based therapies, the overall global burden of HF remains high. Large-scale national reporting initiatives consistently identify HF as a major contributor to cardiovascular morbidity, mortality, and healthcare expenditure, underscoring that the HF burden persists despite therapeutic advances [5].

A key factor underpinning this persistent burden is the increasing prevalence of asymptomatic or unrecognized HF and subclinical left ventricular (LV) dysfunction, collectively termed stage B HF (SBHF). This pattern is largely driven by population aging and the growing burden of hypertension, diabetes mellitus, and cancer, which are strongly associated with an increased risk of HF development. Consequently, preventive or disease-modifying interventions are often delayed or not implemented [6]. This is a problem because treatment of established HF alone is insufficient to meaningfully reduce the population-level burden of the disease. Early identification of individuals at risk and the implementation of preventive strategies during the subclinical HF phases are increasingly recognized as essential approaches to reduce incident HF and its long-term clinical and economic consequences [7]. Importantly, the treatment of subclinical HF has emerged as a potential upstream target in international strategies to mitigate the overall HF burden [8]. However, there remains a knowledge gap pertaining to who, how and when to test for SBHF. Therefore, this review aims to summarize and provide an up-to-date overview of established diagnostic tests and emerging tools for the early detection of SBHF.

## Target Population for the Diagnosis of SBHF

**Fig. 1:**
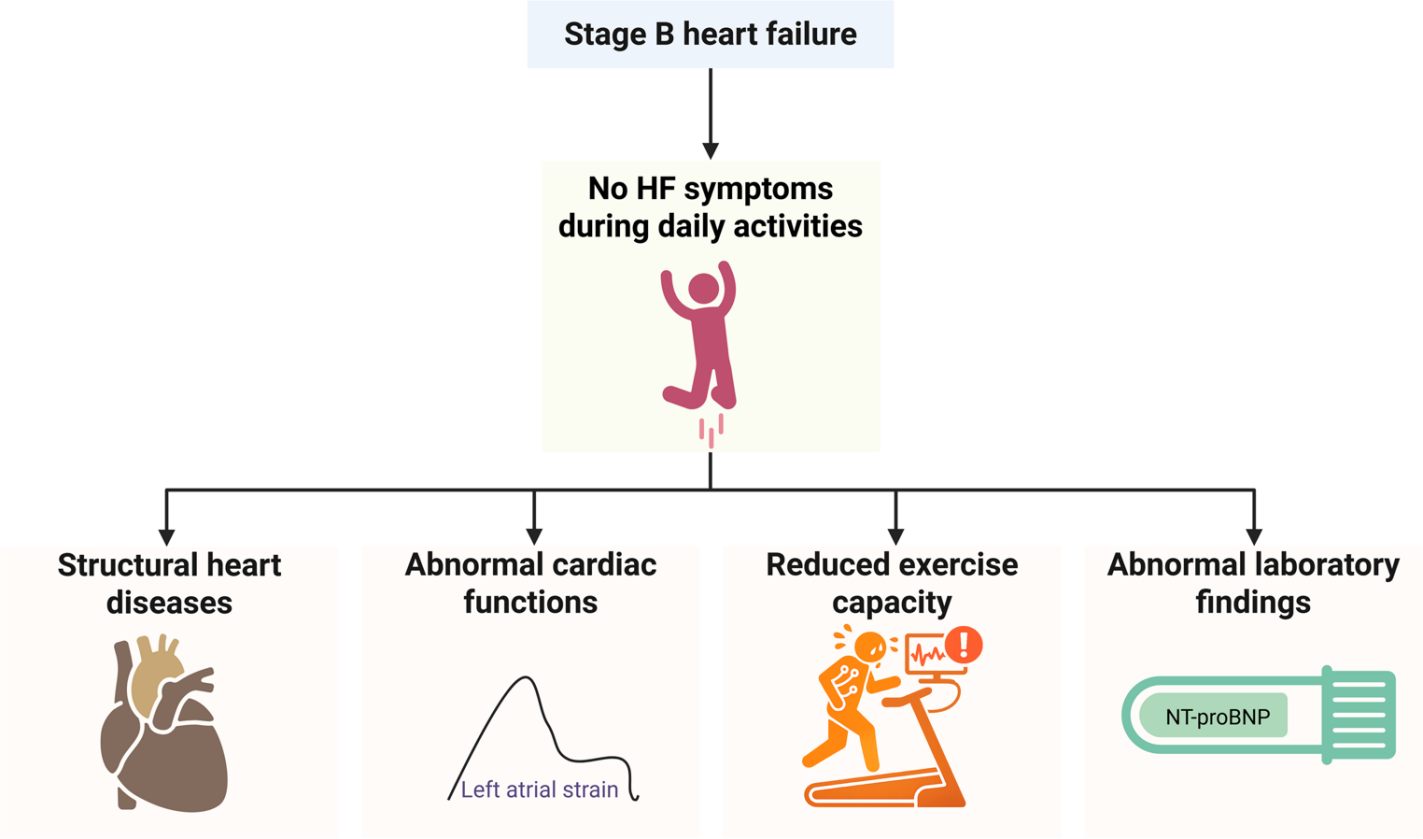
Diagnostic components of SBHF. Created in BioRender. Dang, N. N. H. (2026) https://BioRender.com/jab7rpe

SBHF represents a subclinical phase in the HF continuum, during which pathological myocardial remodelling and/or hemodynamic disturbances are already present, but overt clinical manifestations of HF have not yet developed [3, 9] (Fig. 1). The universal definition of HF recognizes SBHF in the presence of objective evidence of cardiac abnormalities [10]. These abnormalities include at least one of the following domains: structural heart disease, abnormal cardiac function, or persistently abnormal cardiac biomarkers. Cardiac structural changes include LV hypertrophy, cardiac chamber enlargement, regional wall motion abnormalities, myocardial tissue abnormalities, or clinically significant valvular heart disease [3]. In addition to structural alterations, functional abnormalities also fulfil the criteria for SBHF. These include reduced left or right ventricular systolic function, evidence of elevated cardiac filling pressures, or abnormal diastolic function. Furthermore, patients with persistently elevated circulating cardiac biomarkers, particularly natriuretic peptides (NPs) or cardiac troponins, are classified as having SBHF when competing causes of biomarker elevation have been excluded [3, 11].

HF is a clinical diagnosis, but symptoms are inherently subjective and may be misleading—a limitation regarded as the “Achilles’ heel” of SBHF diagnosis [12]. The requirement of the “absence of symptoms” means that SBHF covers two entities—unrecognized HF (the patient does not complain of symptoms because of inactivity or attribution to other causes such as aging or obesity) and truly subclinical impairment of cardiac structure or function. The latter group might be considered “early” SBHF, with echo features but without reduced exercise capacity or elevated natriuretic peptides. In contrast, late SBHF may involve individuals who do not report subjective symptoms but show objective abnormalities. These findings may be identified incidentally or through targeted assessment, such as hospital-based screening or exercise testing.

Universal screening for SBHF is not feasible because of substantial economic constraints. For this reason, a targeted screening approach is more pragmatic [13]. These include individuals with hypertension [14, 15], diabetes mellitus [16, 17], obesity [15, 18], and chronic kidney disease [19, 20], which represent the most common substrates for subclinical HF. Advancing age is strongly associated with SBHF, with a particularly high prevalence among older adults [21–23]. In addition, first-degree relatives of patients with inherited cardiomyopathies remain at increased risk, even in the absence of symptoms [24]. Several additional risk enhancers have been identified. These include prior COVID-19 or HIV infection [25, 26], exposure to cardiotoxic cancer therapies [27, 28], and adverse pregnancy-related outcomes [29]. Lifestyle and social determinants also play a significant role. Smoking, physical inactivity, abnormal sleep duration, social deprivation, rural residence, chronic stress, and air pollution contribute to accelerated HF progression [30–32]. The high-risk populations and supporting evidence are summarized in Table 1.

**Table 1:** High-risk populations prioritized for SBHF screening

High-risk population	Evidence	Rationale for SBHF screening
Older adults	Mureddu et al. [21] Jacobs et al. [22] Ledwidge et al. [23]	Very high subclinical HF prevalence. Aging increases arterial stiffness and volume overload.
Diabetes	Huelsmann et al. [16] Pop-Busui et al. [17]	HF is the most common CV complication. Elevated NP strongly predicts HF hospitalization and mortality.
Hypertension	Lloyd-Jones et al. [14] Aoki et al. [15]	Sustained hypertension markedly increases HF risk. Blood pressure control could reduce incident HF.
Obesity	Aoki et al. [15] Packer et al. [18]	Obesity is associated with a progressive increase in SBHF risk. Excess adiposity suppresses NP levels and may mask early HF.
Smoking	Nadruz et al. [31] Ding et al. [32]	HF risk increases in a dose-dependent manner. Smoking causes subclinical myocardial injury with elevated cardiac biomarkers.
Chronic kidney disease	Agarwal et al. [19] Blecker et al. [20]	Chronic kidney disease confers a high subclinical HF burden. Albuminuria reflects early endothelial dysfunction and subclinical cardiac damage.
Cancer survivors	Soh et al. [27] Ehrhardt et al. [28]	Cancer therapies induce cumulative and long-term cardiotoxicity, leading to progressive myocardial dysfunction.
COVID-19/HIV	Wang et al. [25] Freiberg et al. [26]	COVID-19 is associated with increased risk of new-onset HF. HIV leads to HF development decades earlier than in the general population.
Adverse pregnancy outcomes	Crump et al. [29]	Conditions such as preeclampsia and gestational diabetes are linked to increased long-term HF risk.
Genetic/Family history	Ni et al. [24]	First-degree relatives are at high risk of HF.

Legend – *COVID* coronavirus disease, *CV* cardiovascular, *HF* heart failure, *HIV* human immunodeficiency virus, *NP* natriuretic peptide, *SBHF* stage B HF

## Diagnostic Tests for SBHF

### Initial Clinical Tests

#### Electrocardiography

The simplicity, noninvasiveness, and near universal availability of electrocardiography (ECG) make it an attractive initial tool for cardiovascular assessment in both outpatient and community-based settings, including populations at risk for HF. However, while ECGs remain extensively used in contemporary clinical practice, conventional ECG interpretation has demonstrated limited specificity for the diagnosis and screening of HF, particularly in asymptomatic individuals [33]. These limitations restrict the reliability of conventional ECG as a standalone screening modality for SBHF [34].

The development of ECGs integrated with artificial intelligence (AI) has opened a new avenue to improve the detection of SBHF. Deep learning models applied to standard ECGs are capable of identifying complex signal patterns associated with diastolic dysfunction and structural cardiac remodeling [35]. These models enable the prediction of HF [36], including HF with preserved ejection fraction (HFpEF) [37], with higher diagnostic performance than conventional ECG interpretation [38]. Waveform-based ECG analysis, which incorporates advanced signal processing techniques and machine learning algorithms, allows the extraction of subtle electrocardiographic features that are difficult to detect via visual assessment alone [39]. Emerging evidence suggests that AI-guided ECG analysis, including data derived from single-lead ECGs and wearable devices, can reflect the severity of cardiac dysfunction and the trajectory of subclinical HF progression. This capability enhances early detection of SBHF, including in remote monitoring and population-level screening settings [40, 41].

#### Chest Radiology

Chest X-ray (CXR) has a long history, and its use in routine cardiovascular assessment is based on assessment of cardiac size, pulmonary vasculature, and signs of cardiac decompensation. Importantly, radiographic abnormalities can be present even in patients without overt clinical symptoms, underscoring the potential value of CXR for the early detection of HF [42]. However, CXR has not played a dominant role in the diagnosis or screening of HF and has been superseded by more sensitive imaging techniques [3, 4].

Recently, in parallel with major advances in AI, CXR has regained attention as a potential tool for the early detection of HF, particularly in the preclinical stages. Deep learning models trained on CXR images have demonstrated the ability to capture subtle alterations in cardiac silhouettes and thoracic structures that are difficult for human observers to recognize. An AI-based CXR model can detect severe LV hypertrophy and LV dilation, which are hallmark structural abnormalities of SBHF, with an area under the receiver operating characteristic curve of approximately 0.80. Importantly, the AI model outperformed radiologists in identifying structural cardiac abnormalities from CXR, highlighting the complementary role of AI in augmenting traditional image interpretation [42]. These findings support the potential role of AI-enhanced CXR as a low-cost and widely available modality for the early detection of SBHF, thereby opening new opportunities for HF screening strategies [43].

#### Exercise Stress Testing

Although SBHF describes individuals with HF risk factors who have no current or prior symptoms or signs of HF, a mild objective reduction in functional capacity is common. A symptom questionnaire (e.g., the Duke Activity Status Index) or a 6-minute walk test can provide a simple primary care strategy to identify those at risk and could be used in the selection process for further testing [44].

In SBHF, resting tests may underestimate disease severity because cardiac filling pressures are often normal at rest but become abnormal during physical activity [45]. Exercise stress testing, particularly cardiopulmonary exercise testing combined with bicycle stress echocardiography, helps reveal exercise-related changes in heart function and breathing that indicate early myocardial dysfunction. Long-term studies have shown that exercise test findings such as lower respiratory efficiency, lower exercise capacity, and earlier onset of fatigue, together with exercise-based measures of filling pressure, are associated with a greater risk of future HF hospitalization or cardiac death, even when resting echocardiography results appear normal. These findings support the use of exercise stress testing as a useful tool for early risk assessment and identification of individuals with SBHF, allowing closer follow-up and preventive strategies before symptoms of HF develop [46].

#### Handheld Ultrasound

Recently, handheld ultrasound has emerged as a promising tool to support cardiovascular diagnosis in primary care and remote or resource-limited settings. Its ease of use, bedside availability, portability, and relatively low cost have encouraged clinicians to adopt these devices for rapid medical decision-making. As a result, the use of handheld echocardiography is increasing even among nonexpert operators [47]. A large systematic review and meta-analysis including more than 6,000 participants demonstrated that handheld ultrasound devices have high diagnostic accuracy for detecting LV structural and functional abnormalities. These findings provide strong support for the use of handheld ultrasound devices as an adjunct to physical examination in secondary care, particularly to aid in clinical decision-making when considering referral for standard transthoracic echocardiography [48]. Accordingly, handheld ultrasound represents an effective screening and decision-support tool for suspected HF patients in primary care, especially when combined with telemedicine. However, automated quantitative tools should not be relied upon in the absence of adequate training [49]. More recently, advances in AI have further expanded the potential of handheld echocardiography. Fully automated AI analysis of handheld images has demonstrated high diagnostic accuracy and interchangeability with expert-reported cart-based transthoracic echocardiography for detecting reduced LV ejection fraction (LVEF) in real-world populations with suspected HF. This finding supports its role in rapid triage and exclusion of overt systolic dysfunction [50]. In parallel, AI models applied to handheld ultrasound video clips have shown substantial agreement with standard transthoracic echocardiography in identifying HFpEF, suggesting a future role for AI-enabled handheld ultrasound devices in screening more complex SBHF phenotypes, although confirmatory comprehensive echocardiography remains necessary in ambiguous cases [51].

### Cardiovascular Imaging

#### Echocardiography

Transthoracic echocardiography is consistently regarded as the central diagnostic tool in HF evaluation across international guidelines [3, 9] and is indispensable in SBHF [3].

##### Standard Echocardiography

**Fig. 2:**
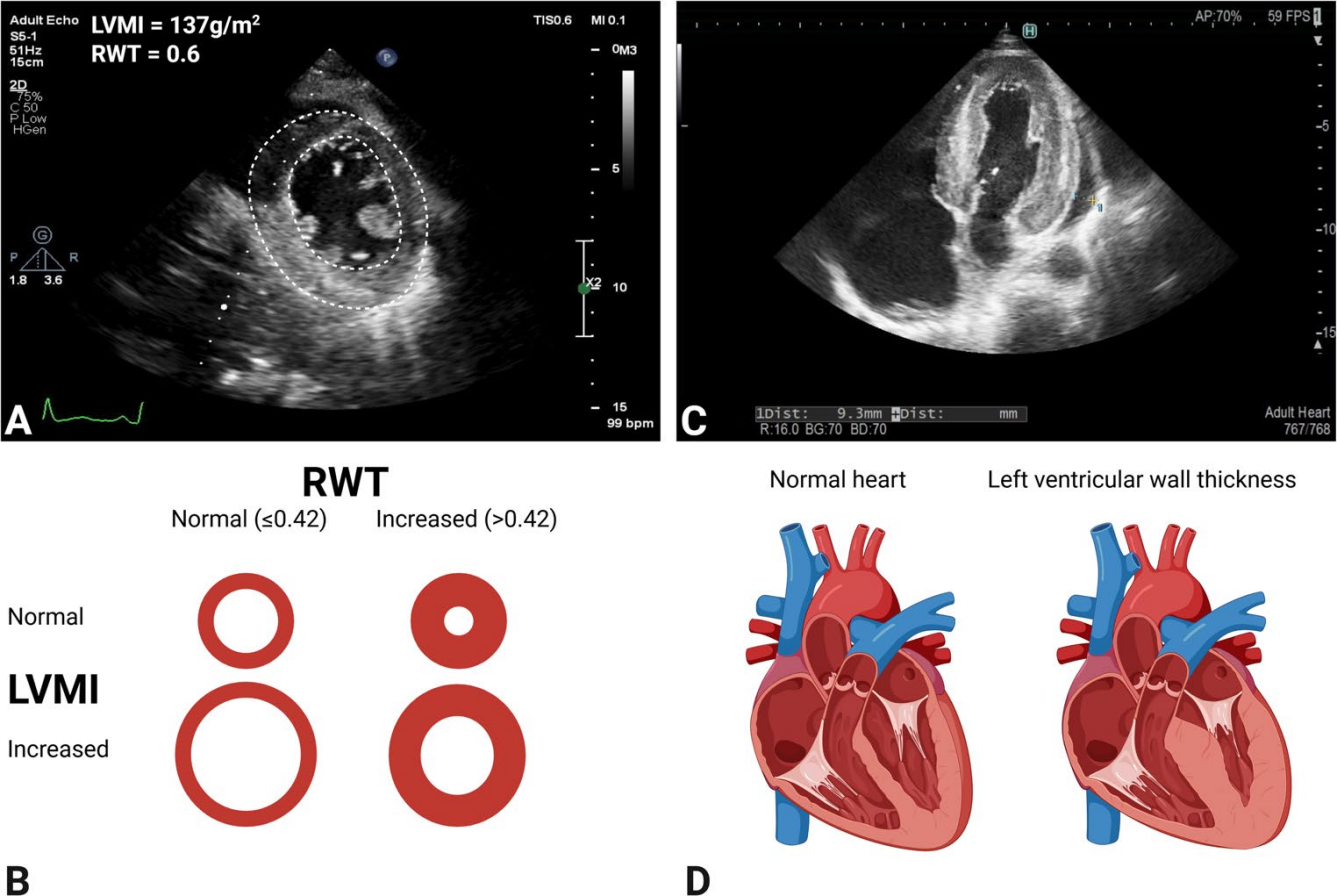
Two-dimensional echocardiographic images of patients with left ventricular hypertrophy. Panel **A**: Parasternal short-axis view on echocardiography. Panel **B**: Different phenotypes of left ventricular hypertrophy. Panel **C**: Apical four-chamber view of a patient with cardiac amyloidosis on echocardiography. Panel **D**: Anatomical myocardial hypertrophy. Abbreviations: LVMI, left ventricular mass index; RWT, relative wall thickness. Created in BioRender. Dang, N. N. H. (2026) https://BioRender.com/7lclcb1

The evaluation of cardiac structure, including the LV mass index, wall thickness, and chamber volume [52], is a fundamental role of echocardiography in SBHF. Structural phenotypes such as concentric remodelling, concentric hypertrophy, eccentric hypertrophy, or early LV dilatation are typical findings in SBHF and are independently associated with future HF development and adverse cardiovascular outcomes (Fig. 2) [53].

**Fig. 3:**
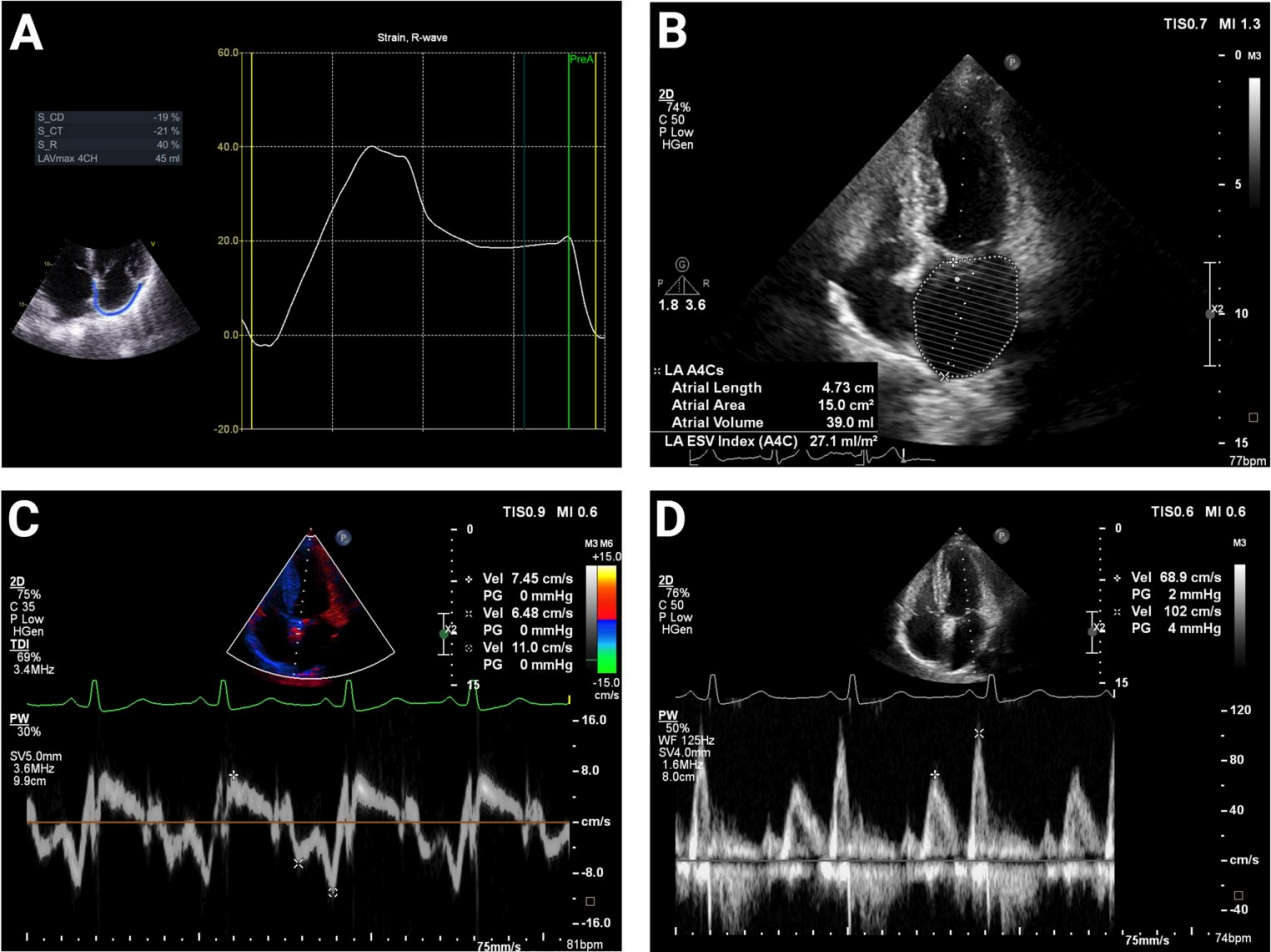
Parameters for the assessment of left ventricular diastolic dysfunction. Panel **A**: Left atrial strain. Panel **B**: Assessment of left atrial volume index (LAVI). Panel **C**: Tissue Doppler imaging–derived early diastolic mitral annular velocity (e′). Panel **D**: Pulsed-wave Doppler assessment of transmitral inflow velocities

The left atrium (LA) warrants particular attention in SBHF. LA enlargement, quantified by indexed LA volume, reflects the cumulative burden of chronically elevated LV filling pressures and frequently develops before overt HF symptoms appear [54]. LA remodelling therefore serves as an integrated marker of long-standing diastolic dysfunction and has strong prognostic value even in asymptomatic populations [55]. Assessment of LV diastolic function is a cornerstone of echocardiographic evaluation in SBHF, and diastolic dysfunction often represents the earliest functional abnormality in subclinical HF and may be present despite preserved LV systolic function (Fig. 3) [56]. Updated recommendations emphasize that intermediate phenotypes are frequently encountered in asymptomatic individuals. Therefore, the assessment of diastolic function should be performed carefully and individually to accurately identify patients who fall within the grey zone. This approach is particularly relevant in SBHF, where early abnormalities may not yet fulfil diagnostic criteria for overt HFpEF but nonetheless indicate increased risk [54]. In addition, echocardiography allows comprehensive evaluation of valvular heart disease, which may drive myocardial remodelling long before symptom onset [57].

##### Three-dimensional Echocardiography

Three-dimensional echocardiography improves the accuracy and reproducibility of chamber volume and LV mass measurements by reducing geometric assumptions and plane dependency [58]. Current chamber quantification guidelines endorse the use of three-dimensional echocardiography when image quality and operator expertise permit, particularly for serial follow-up and risk stratification [59]. However, widespread adoption remains limited by equipment availability and operator experience.

##### Speckle-tracking Echocardiography

**Fig. 4:**
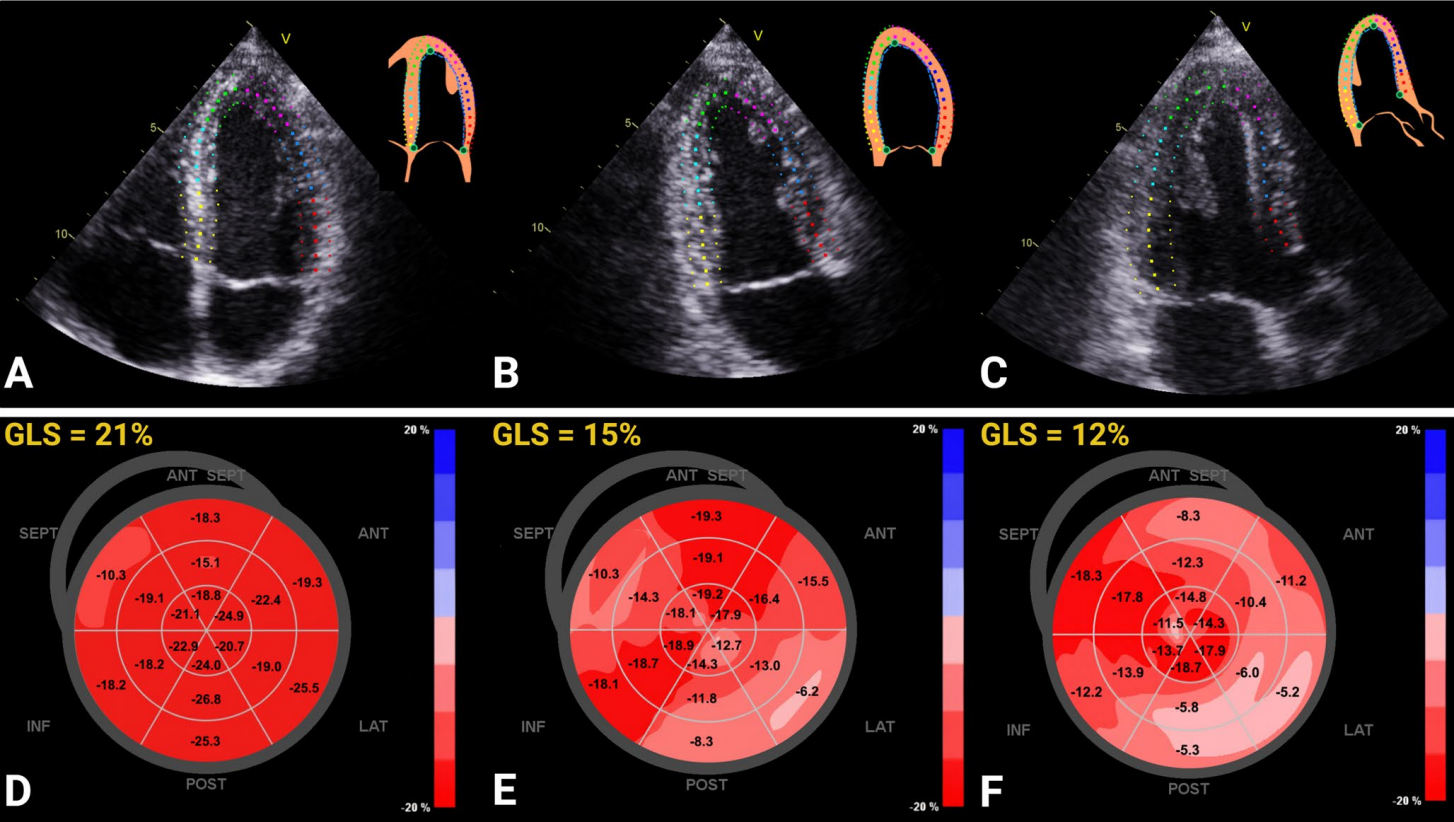
Speckle tracking echocardiography. Panels **A**–**C**: Speckle tracking analysis in different echocardiographic views. Panel **D**: Normal global longitudinal strain (GLS). Panel **E**: Mildly reduced GLS in a patient with heart failure with preserved ejection fraction (HFpEF). Panel **F**: Reduced GLS in a patient with heart failure with reduced ejection fraction (HFrEF)

Speckle tracking echocardiography has emerged as a key modality for identifying subclinical myocardial dysfunction in individuals with SBHF [60]. LV global longitudinal strain (GLS) enables the detection of early systolic impairment even when the LVEF is preserved (Fig. 4) [61]. Population-based studies have demonstrated that impaired GLS, in combination with increased LV mass, independently predicts incident HF in asymptomatic individuals, supporting its role as a sensitive marker of SBHF [62–64].

LA strain provides functional information beyond LA size and reflects atrial compliance and reservoir function. Recent guidelines recognize LA strain as a valuable adjunct in diastolic assessment, particularly for estimating LV filling pressures and identifying early HFpEF pathways (Fig. 3A) [54, 65]. Patients with SBHF have been shown to exhibit reduced LA reservoir strain, indicating early atrial dysfunction before clinical HF becomes apparent [66, 67].

##### Echocardiography Integrated with Artificial Intelligence

Despite its diagnostic strength, echocardiography remains highly operator dependent, requiring substantial expertise in image acquisition, measurement, and interpretation [68]. This limitation constitutes a major barrier to the widespread application of echocardiography for population-level screening of SBHF.

AI may facilitate both image acquisition and interpretation by nonexperts. Real-time deep learning-based guidance for image acquisition by less experienced operators has been shown to improve the standardization of echocardiographic examinations [69]. This approach may increase the number of personnel capable of performing echocardiography while simultaneously enhancing both the quantity and quality of measurements [70]. AI-enhanced interpretation is based on automated image analysis, the extraction of quantitative parameters, the management of missing data, and the generation of probability-based risk outputs [71]. AI-enabled echocardiographic models have demonstrated promising performance in identifying LV dysfunction and prognostically relevant phenotypes using routine echocardiographic data, even when conventional parameters appear normal [72]. By reducing interobserver variability and technical dependence, AI has the potential to transform echocardiography from a specialist-centered modality into a scalable screening tool for early HF detection.

##### Challenges in Using Echocardiography for SBHF Screening

Although echocardiography provides rich diagnostic information, its use for SBHF screening remains inconsistent. Key barriers include operator dependency, time and workflow constraints, lack of standardized reporting, and uncertainty regarding optimal screening populations. In addition, the number of trained echocardiography specialists remains insufficient to meet the growing demand for cardiac imaging in many healthcare systems [73]. Current evidence suggests that targeted screening of high-risk individuals [74], combined with standardized imaging protocols, structured reporting systems, and AI augmentation, may be a feasible strategy for expanding echocardiographic detection of SBHF without compromising diagnostic quality.

#### Cardiac Magnetic Resonance Imaging

Cardiac magnetic resonance (CMR) is uniquely suited for characterizing myocardial abnormalities at both the macroscopic and tissue levels, thereby refining risk stratification and improving the mechanistic understanding of this early phase of HF [75]. CMR is regarded as the reference standard for the assessment of ventricular volume, myocardial mass, and ejection fraction because of its high spatial resolution and excellent reproducibility [76]. These attributes are especially relevant in SBHF, where subtle LV remodelling, such as early LV dilatation or concentric hypertrophy, may carry important prognostic implications yet remain difficult to quantify reliably via other imaging modalities. CMR enables precise phenotyping of asymptomatic LV remodelling, allowing early identification of individuals who meet the SBHF criteria before progression to symptomatic disease [77]. In addition, CMR-derived measurements of LV mass and geometry provide robust markers of pressure or volume overload states commonly encountered in hypertension, diabetes mellitus, and ischaemic heart disease [78].

**Fig. 5:**
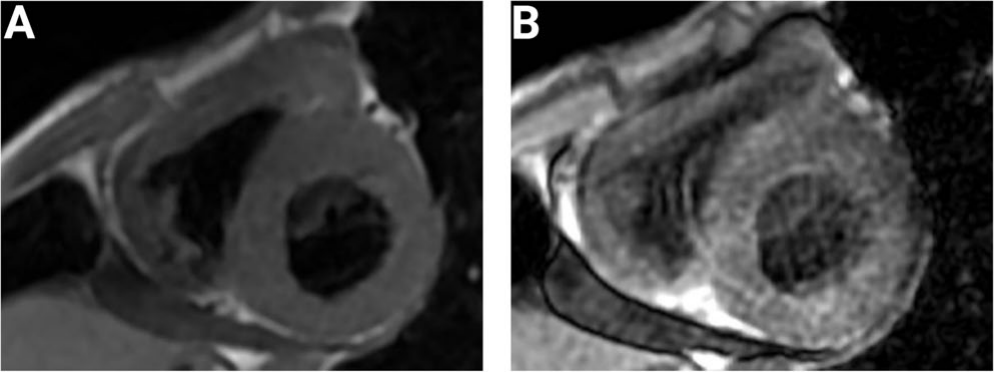
Cardiovascular magnetic resonance imaging of a patient with cardiac amyloidosis. Panel **A**: Pre-contrast image. Panel **B**: Late gadolinium enhancement (LGE) image

A defining strength of CMR in SBHF is its ability to provide noninvasive myocardial tissue characterization with late gadolinium enhancement (Fig. 5). The detection of replacement fibrosis facilitates the identification of silent myocardial infarction, non-ischemic scar patterns, or infiltrative cardiomyopathies in asymptomatic individuals [79]. Myocardial fibrosis is also a key pathophysiological substrate for progression to overt HF and ventricular arrhythmias, even in the absence of clinical symptoms [75]. In SBHF, the presence, distribution, and extent of late gadolinium enhancement provide important etiologic and prognostic information that may directly influence preventive and therapeutic strategies. Parametric mapping techniques, including native T1 mapping and extracellular volume quantification, enable the quantitative assessment of diffuse interstitial fibrosis [80]. This myocardial abnormality, related to cardiometabolic disease and pressure overload, may appear before conventional structural changes become evident [81]. In SBHF, elevated native T1 or extracellular volume values may reflect early diffuse interstitial fibrosis and extracellular matrix expansion that precede symptomatic HF, supporting their role as early imaging biomarkers of disease progression.

Abnormal myocardial deformation is increasingly recognized as a functional hallmark of SBHF, particularly in patients with diabetes mellitus and a preserved ejection fraction [67, 82, 83]. CMR-based myocardial strain analysis, which is most commonly performed via feature tracking, allows sensitive detection of subclinical myocardial dysfunction. Although CMR-derived strain benefits from superior image quality and reduced dependency on acoustic windows compared with echocardiography [84], imaging has a lower temporal resolution. Despite its strengths, CMR is limited by higher cost, lower availability, longer scan times, and the need for specialized expertise [85]. These factors limit its feasibility as a universal screening modality for SBHF. CMR complements rather than replaces echocardiography by providing incremental diagnostic and prognostic value in selected settings, particularly when echocardiographic findings are inconclusive or when detailed myocardial tissue characterization is clinically relevant [77].

#### Cardiac Computed Tomography

The primary value of cardiac computed tomography (CT) in the assessment of SBHF lies in etiologic clarification and assessment of structural substrates. Coronary CT angiography is particularly useful for excluding occult coronary artery disease in asymptomatic individuals with LV remodelling or subclinical dysfunction, thereby enabling early identification of ischaemic phenotypes [86]. Cardiac CT also allows accurate quantification of the LV mass, chamber volume, and LA size when echocardiographic assessment is limited or inconclusive [87]. Emerging techniques, such as CT-derived extracellular volume quantification, permit indirect assessment of diffuse myocardial fibrosis in patients who are unsuitable for CMR [88]. In addition, CT can detect subclinical pulmonary congestion in patients with HF, contributing to earlier diagnosis and improved risk stratification [89]. However, owing to radiation exposure and/or the need for iodinated contrast, cardiac CT is not appropriate as a screening modality [90]. Instead, it should be considered a targeted adjunct within a multimodality imaging strategy for selected patients with SBHF.

#### Nuclear Imaging

In SBHF, nuclear imaging serves as a selective adjunct modality for identifying subclinical myocardial abnormalities that precede HF. Myocardial perfusion imaging enables the detection of ischemia, scarring, and viability. These capabilities help distinguish ischemic from nonischemic substrates underlying asymptomatic structural heart disease [91]. Advanced positron emission tomography techniques allow quantitative assessment of myocardial blood flow and coronary flow reserve, providing incremental risk stratification in patients with early ventricular remodelling or cardiomyopathy [92]. In addition, cardiac sympathetic innervation imaging using iodine-123 metaiodobenzylguanidine can detect early autonomic dysfunction, which is a pathophysiological feature associated with adverse remodelling and progression toward symptomatic HF [91]. While contemporary multimodality imaging frameworks emphasize that nuclear techniques retain a selective role in early HF phenotyping [93], their main roles are etiologic clarification and prognostic assessment rather than routine screening [87].

### Biomarkers

Biomarkers are used for three purposes in SBHF. They can be used in screening to identify high-risk individuals for referral to cardiac imaging. If imaging is used for screening, it can be used to support the diagnosis of SBHF when imaging results are equivocal. Finally, they can identify silent myocardial injury or stress that precedes overt HF [11, 94].

#### Natriuretic Peptides

B-type natriuretic peptide (BNP) and N-terminal pro-B-type natriuretic peptide (NT-proBNP) are the most consistently validated circulating biomarkers for predicting incident HF. The attraction is that they are readily measured and that the numbers are easily scaled. However, as they reflect myocardial wall stress, they may be more effective in the detection of unrecognized HF than preclinical HF. Large multicohort proteomic analyses have shown that NPs remain the strongest individual predictors of future HF, and adding a multiprotein biomarker approach usually provides only modest improvement beyond that of NPs alone [95].

In risk-based screening strategies, particularly in high-risk cardiometabolic populations, NP-guided pathways are emphasized as a practical approach to identify subclinical cardiac dysfunction at an earlier stage [96]. In SBHF, there are no specific NP cut-offs that independently establish the diagnosis. Instead, BNP ≥ 35 pg/mL or NT-proBNP ≥ 125 pg/mL in the nonacute setting are widely accepted as indicators of abnormal myocardial stress and are used for risk enrichment and screening triage. Values above these thresholds, but below rule-in levels for overt HF, are frequently observed in asymptomatic individuals with early structural or functional cardiac abnormalities and are associated with an increased risk of future HF. Therefore, NPs should be interpreted in conjunction with cardiac imaging to support the identification of SBHF rather than being used in isolation [97].

#### Troponin

High-sensitivity cardiac troponin (hs-cTn) reflects ongoing low-grade myocardial injury and is used for risk stratification and, in some settings, screening enrichment in asymptomatic high-risk populations. Recent reviews highlight hs-cTn as a strong candidate biomarker for predicting future HF, especially when it is combined with clinical risk factors and other biomarkers [98]. In large population-based analyses of individuals without baseline HF, myocardial infarction, or stroke, sex-specific hs-cTnI thresholds have been proposed to identify individuals at increased future risk of HF. Values of hs-cTnI ≥ 2.6 ng/L in women and ≥ 4.2 ng/L in men were shown to best discriminate higher risk groups and were intended for preventive risk targeting rather than confirmation of established HF. Importantly, these thresholds are assay dependent and should not be interpreted as universal diagnostic cut-offs [99].

For hs-cTnT, low-level detectability is common even in Stages A HF and SBHF. The risk associated with hs-cTnT appears to increase in a continuous manner, making the application of a single universal threshold challenging. The commonly used 99th percentile upper reference limit of approximately 14 ng/L for one widely used assay was derived from relatively young reference populations and may underestimate normal values in older individuals, particularly men. Therefore, interpretation of hs-cTnT in SBHF should account for age, sex, and assay-specific characteristics rather than relying on a rigid cut-off [100]. Accordingly, in SBHF, hs-cTn should be interpreted as a marker of subclinical myocardial injury and disease trajectory rather than a diagnostic criterion. Its greatest value lies in combination with clinical risk factors, NPs, and cardiac imaging to identify individuals who may benefit from closer surveillance or preventive interventions.

#### Emerging Biomarkers

Large-scale proteomic studies have identified groups of circulating biomarkers involved in myocardial fibrosis and adverse remodelling that are associated with incident HF before the onset of clinical symptoms [95]. Among these biomarkers, soluble suppression of tumorigenicity 2 reflects myocardial stress and fibrosis signaling [101]. Galectin-3 is linked to inflammation-driven remodelling of the extracellular matrix during the transition from subclinical cardiac disease to overt HF [102]. These biomarkers are involved in extracellular matrix turnover, vascular dysfunction, and inflammatory remodelling. These processes represent central mechanisms in the progression of SBHF. Beyond structural remodelling, proteomic profiling in community-based populations has shown that biomarkers predicting new-onset HF often cluster within systemic inflammatory- and apoptosis-related pathways. These findings support the concept that the progression from SBHF to symptomatic HF is driven not only by mechanical stress but also by immunometabolic processes. However, limited incremental value beyond NPs and challenges related to standardization, cost, and clinical implementation currently restrict the widespread use of these approaches [103].

In parallel, the urine albumin-to-creatinine ratio (uACR) is a traditional laboratory test that is increasingly recognized as a risk marker for HF and may help identify patients who could benefit from targeted treatment strategies known to reduce HF risk [104]. The uACR plays an important role in the identification, risk stratification, and management of SBHF. In a large prospective cohort study including more than 9,000 patients, an elevated uACR was shown to be strongly and dose-dependently associated with incident HF, even at levels below the conventional threshold for microalbuminuria. Compared with individuals with a normal uACR, the risk of HF increased by more than twofold in those with microalbuminuria and by approximately sixfold in those with macroalbuminuria after multivariable adjustment [105]. These findings support the use of the uACR as a powerful tool for identifying individuals at high risk of progression to overt, symptomatic HF, thereby reinforcing its clinical value in SBHF detection and prevention strategies [106].

#### Scoring Systems and Artificial Intelligence

SBHF is a heterogeneous and difficult-to-identify clinical entity because patients lack overt symptoms, whereas structural abnormalities, functional changes, and biomarker elevations are often subtle. Many test results fall within borderline or gray zone ranges rather than clearly exceeding diagnostic thresholds. This further complicates the diagnosis. As a result, increasing attention has been given to multivariable models and composite scoring systems designed to improve diagnostic certainty, risk stratification, and functional characterization of SBHF beyond the assessment of individual parameters. These models typically integrate clinical data, echocardiographic findings, and circulating biomarkers. This approach reflects the multimechanical nature of preclinical HF [107].

**Fig. 6:**
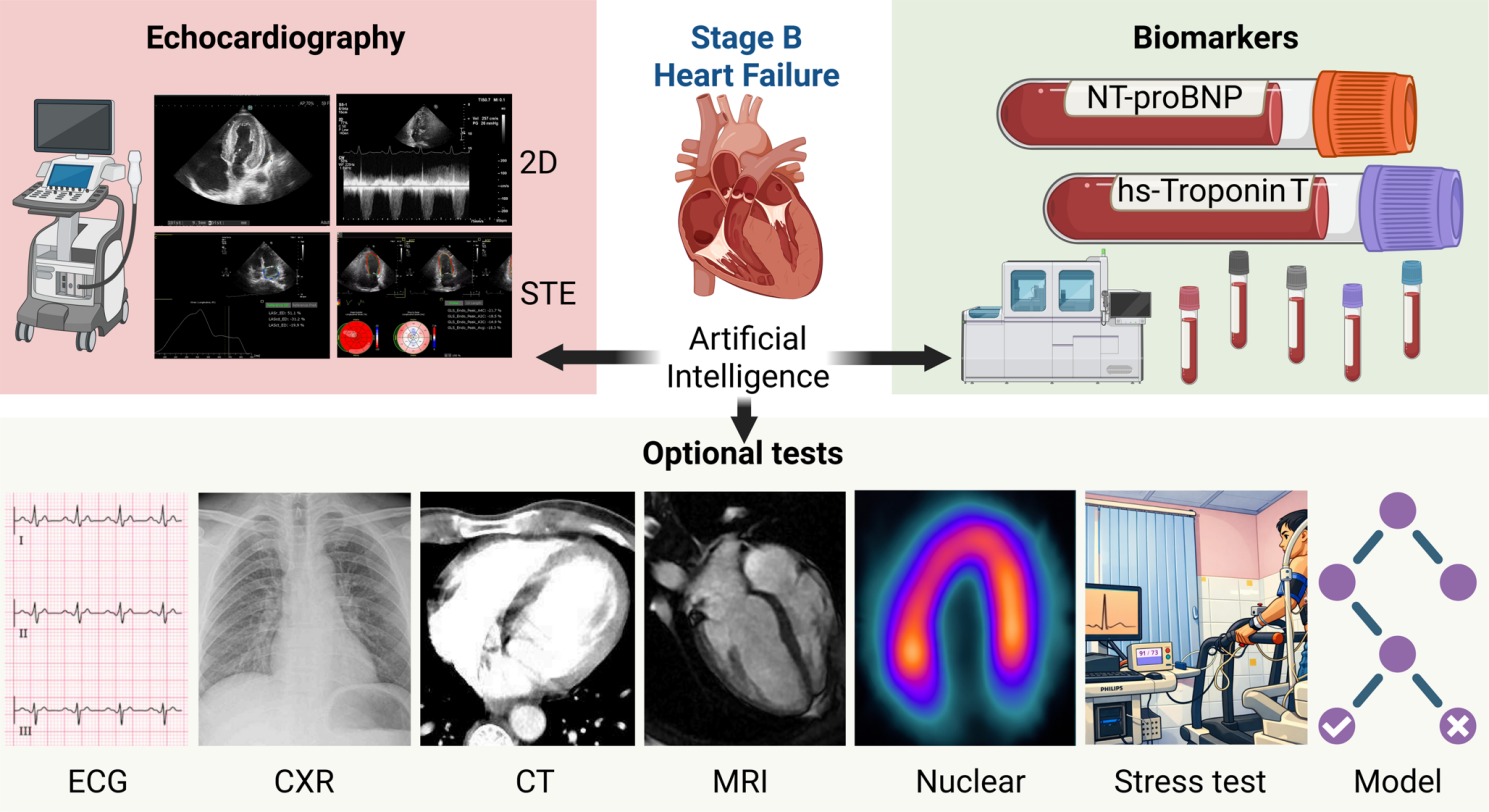
Overview of diagnostic methods for Stage B heart failure. Echocardiography and biomarkers are the primary diagnostic components. Artificial intelligence provides supportive tools to improve the precision of these tests. Abbreviations: HF, Heart failure; 2D, Two-dimensional echocardiography; STE, Speckle tracking echocardiography; ECG, Electrocardiography; CXR, Chest X-ray; CT, Cardiac computed tomography; MRI, Cardiac magnetic resonance imaging. Created in BioRender. Dang, N. N. H. (2026) https://BioRender.com/dbab5kc

At present, no model or scoring system has been specifically validated for the diagnosis of SBHF. However, rapid advances in AI and machine learning have enabled the development of data-driven models using large-scale echocardiographic datasets and electronic health records [108]. These models aim to detect preclinical patterns of cardiac remodelling and offer a promising direction for earlier identification of SBHF in future clinical practice [107]. Figure 6 summarizes the key diagnostic modalities used for the identification of SBHF.

## Where, When, and How Often Should Screening for SBHF be Performed?

Primary care represents the most cost-effective setting for baseline risk stratification and for identifying individuals who require escalation to cardiac imaging or specialist referral. High-risk populations should therefore be screened at this level via a clinical risk assessment and NPs. The use of NP testing and handheld ultrasound integrated with AI is increasingly promoted to improve accessibility. Evidence from the STOP-HF trial demonstrated that NP–guided screening in primary care, followed by collaborative cardiology care, significantly reduced the incidence of HF and asymptomatic LV dysfunction [23].

**Fig. 7:**
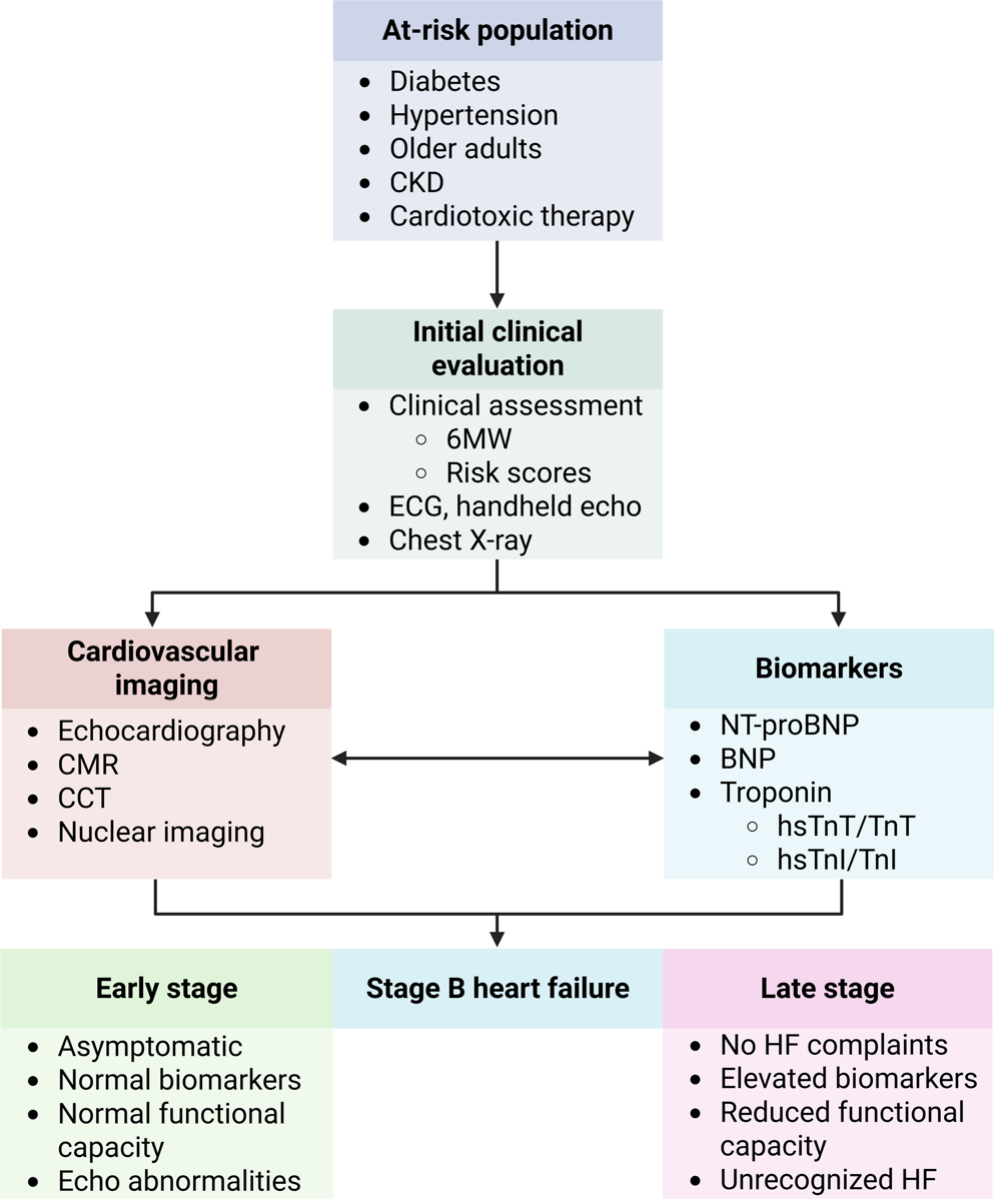
Diagnostic flowchart for SBHF. Abbreviations: HF, heart failure; CKD, chronic kidney disease; ECG, electrocardiography; 6 MW, six-minute walk test; CXR, chest X-ray; CMR, cardiac magnetic resonance imaging; CCT, cardiac computed tomography; BNP, B-type natriuretic peptide; NT-proBNP, N-terminal pro-B-type natriuretic peptide; hsTnT, high-sensitivity cardiac troponin T; TnT, cardiac troponin T; hsTnI, high-sensitivity cardiac troponin I; TnI, cardiac troponin I

Specialist assessment allows comprehensive evaluation with advanced diagnostic tools and confirmation of SBHF. Importantly, clinicians from other specialties should remain vigilant in identifying high-risk patients and collaborate with cardiologists for further SBHF evaluation. Outpatient specialty clinics, including cardiology, endocrinology for diabetes, and nephrology services, are key settings for screening high-risk groups. Dedicated disease management units, such as oncology centers for patients exposed to cardiotoxic chemotherapy and HIV clinics, also represent important screening environments [26, 27]. Screening intervals should be individualized according to the specific risk profile. Annual screening via NP testing may be considered in high-risk individuals without established structural heart disease [104]. In cancer survivors, screening is advised at 3 months and 12 months after the completion of chemotherapy during the first year [109]. The diagnostic workflow for SBHF is summarized in Fig. 7.

## Conclusion

At present, multiple diagnostic modalities are available for the evaluation of SBHF. However, echocardiography and circulating biomarkers remain the core diagnostic components, whereas other tests serve complementary roles. The integration of AI into these diagnostic modalities helps simplify and enhance the detection of SBHF in clinical practice.

## Key References

- Ledwidge M et al. Natriuretic Peptide–Based Screening and Collaborative Care for Heart Failure: The STOP-HF Randomized Trial. *JAMA*. 2013;310(1):66-74.
  - ○ This RCT is a cornerstone of the evidence that shows the impact of diagnosis and management of stage B heart failure. The intervention group (BNP-driven collaborative care) had a lower incidence of LV dysfunction and heart failure than the controls.
- Hauptmann T et al. Echocardiographic measures read by artificial intelligence enable accurate and rapid prediction of the worsening of heart failure. *European Heart Journal - Digital Health*. 2025;6(6):1246-1256.
  - ○ This recent paper shows the impact of AI interpretation of echo in a community-based screening process. In 2466 patients followed over a median of 3.8 years, 470 experienced worsening of heart failure. There was better performance for automatically determined LVEF (0.71 vs. 0.73, *P* = 0.038) and *E/E′*-ratio (0.64 vs. 0.66, *P* = 0.013) and a trend for LVM (0.66 vs. 0.68, *P* = 0.063) over standard reads.
- Lafitte S et al. Integrating artificial intelligence into an echocardiography department: Feasibility and comparative study of automated versus human measurements in a high-volume clinical setting. *Archives of Cardiovascular Diseases*. 2025;118(8-9):477-488.
  - ○ There is an understandable emphasis in the literature about the development and validation of AI, but less about its use in practice. This paper showed good agreement between AI and human measurements for EF (ICC=0.81) with a global mean difference of -4% (SD 15%).

## Supplementary Information

Below is the link to the electronic supplementary material.

Supplementary Material 1

Supplementary Material 2

Supplementary Material 3

## Data Availability

No datasets were generated or analysed during the current study.
