# Supplementary material for: Diagnostic Tests for Stage B Heart Failure
Source: Curr Cardiol Rep. 2026 Jul 4;28(1):65. doi: 10.1007/s11886-026-02389-x (PMC13331820; doi:10.1007/s11886-026-02389-x)
Supplement: Supplementary file 3 — Supplementary Material 3 [file 11886_2026_2389_MOESM3_ESM.pdf]

49 Spadina Ave. Suite 200
Toronto ON M5V 2J1 Canada
www.biorender.com

Confirmation of Publication and Licensing Rights

April 16th, 2026

Subscription Type:	*Student Plan - Academic*
Agreement number:	*AT29LR3UCU*
Publisher Name:	*Current Cardiology Reports*
Citation to Use:	*Created in BioRender. Dang, N. N. H. (2026) https://BioRender.com/dbab5kc*

To whom this may concern,

This document is to confirm that Hai Nguyen Ngoc Dang has been granted a license to use the BioRender Content, including icons, templates, and other original artwork, appearing in the attached Completed Graphic pursuant to BioRender's Academic License Terms. This license permits BioRender Content to be sublicensed for use in publications (journals, textbooks, websites, etc.).

All rights and ownership of BioRender Content are reserved by BioRender. All Completed Graphics must be accompanied by the following citation: "Created in BioRender. Dang, N. N. H. (2026) https://BioRender.com/dbab5kc".

BioRender Content included in the Completed Graphic is not licensed for any commercial uses beyond use in a publication. For any commercial use of this figure, users may, if allowed, recreate it in BioRender under an Industry BioRender Plan.

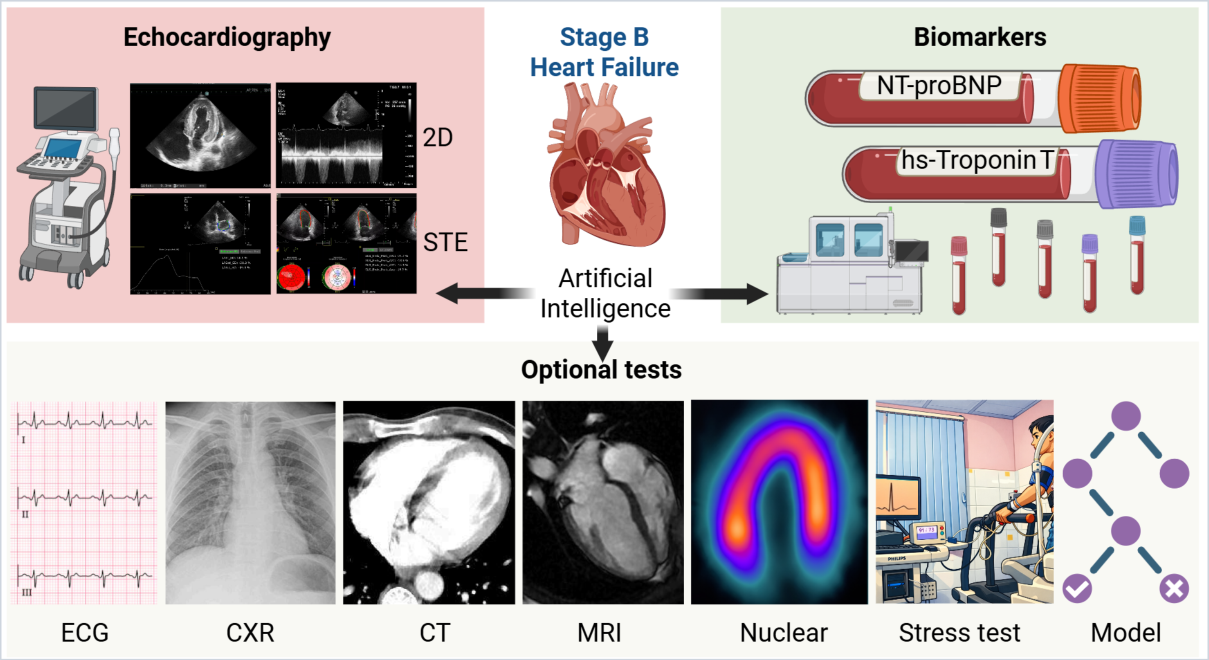

For any questions regarding this document, or other questions about publishing with BioRender, please refer to our BioRender Publication Guide, or contact BioRender Support at support@biorender.com.
